# Detection of central visual field defects in early glaucomatous eyes: Comparison of Humphrey and Octopus perimetry

**DOI:** 10.1371/journal.pone.0186793

**Published:** 2017-10-27

**Authors:** Gloria Roberti, Gianluca Manni, Ivano Riva, Gabor Holló, Luciano Quaranta, Luca Agnifili, Michele Figus, Sara Giammaria, Davide Rastelli, Francesco Oddone

**Affiliations:** 1 IRCSS-Fondazione GB Bietti, Rome, Italy; 2 DSCMT, University of Rome Tor Vergata, Rome, Italy; 3 Department of Ophthalmology, Semmelweis University, Budapest, Hungary; 4 Section of Ophthalmology, Department of Medical and Surgical Specialties, Radiological Sciences, and Public Health University of Brescia, Brescia, Italy; 5 Ophthalmology Clinic, Department of Medicine and Aging Science, "G. d'Annunzio" University of Chieti-Pescara, Chieti, Italy; 6 Department of Neurosciences, University of Pisa, Pisa, Italy; 7 University of Rome Campus Biomedico, Rome, Italy; Bascom Palmer Eye Institute, UNITED STATES

## Abstract

**Purpose:**

To compare the detection rate of central visual field defect (CVFD) between the 30-degree Octopus G1 program (Dynamic strategy) and the HFA 10–2 SITA-Standard test in early glaucoma eyes not showing any CVFD on the HFA 24–2 SITA-Standard test.

**Methods:**

One eye of 41 early glaucoma patients without CVFD in the central 10° on HFA 24–2 test was tested with both the HFA 10–2 test and the Octopus G1 program 15 minutes apart, in random order. The primary outcome measure was the comparison of CVFD detection rates. Secondary outcome measures comprised the agreement in detecting CVFD, and the comparison of test durations and the numbers of depressed test points outside the central 10-degree area between the HFA 24–2 test and the Octopus G1 program.

**Results:**

The mean age of the population was 65.2±10.1 years, and the mean deviation with HFA 24–2 was -3.26±2.6 dB. The mean test duration was not significantly different between the tests (p = 0.13). A CVFD was present in 33 (80.4%) HFA 10–2 test and in 23 (56.0%) Octopus G1 tests (p = 0.002). The overall agreement between the HFA 10–2 and Octopus G1 examinations in classifying eyes as having or not having CVFD was moderate (Cohen’s kappa 0.47). The Octopus G1 program showed 69.6% sensitivity and 100% specificity to detect CVFD in eyes where the HFA 10–2 test revealed a CVFD. The number of depressed test points (p<5%) outside the central 10° area detected with the Octopus G1 program (19.68±10.6) was significantly higher than that detected with the HFA 24–2 program (11.95±5.5, p<0.001).

**Conclusion:**

Both HFA 10–2 and Octopus G1programs showed CVFD not present at HFA 24–2 test although the agreement was moderate. The use of a single Octopus G1 examination may represent a practical compromise for the assessment of both central and peripheral visual field up to 30° eccentricity without any additional testing and increasing the total investigation time.

## Introduction

Glaucoma is one of the leading causes of irreversible blindness [1].

It is characterized by the loss of retinal ganglion cells (RGC) and the corresponding typical visual field defects [2]. Recent studies showed that glaucomatous changes in the central visual field may already be present in the early stage of the disease, which is consistent with the results of imaging studies [3–6]. Thus, detection of early glaucomatous visual field changes at any eccentricity is important to successful glaucoma detection and management. The 24–2 SITA test of the Humphrey Field Analyzer (HFA, Carl Zeiss Meditec, Dublin, CA) is one of the most frequently used tests in clinical practice when normal and glaucomatous eyes need to be separated [7]. This fast test employs a grid of 54 test locations evenly distributed with 6° separation. Twelve of the 54 test-point locations are in the central 10°. Of them 4 locations cover the central 8° area. However, more than 30% of the retinal ganglion cells reflect the function of this area [8,9], thus in the HFA 24–2 test the sampling of the central visual field area may be underpowered. There is a wide agreement between researchers and clinicians that the low spatial resolution of this program in the central macular representation might be a major factor of the underestimation of functional deterioration in glaucoma with this system, independently from the stage of the disease [10–15].

The central visual field area can be selectively and more accurately tested using the HFA 10–2 test which employs a test-point grid of higher spatial resolution for the assessment of the central 10-degree visual field area. It has 68 test point locations evenly distributed with 2° separation in the central 10-degree [8]. Recently it has been shown that the HFA 10–2 program makes it possible to detect central visual field defects (CVFD) which are not detected with the HFA 24–2 program already in early glaucoma [10–15]. However, the HFA 10–2 test does not investigate the peripheral visual field outside the central 10-degree area, thus the patients need to perform both the 10–2 and the 24–2 tests for optimal decision making, which unfavorably increases the overall testing time.

In contrast, the G1 program of the Octopus perimeter (Haag-Streit AG, Koeniz-Berne, Switzerland) employs an unevenly distributed grid of 59 test locations within the 30°, in which the test-point density is higher nasally than temporally, and around the macula than in the more peripheral areas. The Octopus G1 program has 5 central points with 2.8° separation for the fovea representation, and 17 test locations for the macula. This test point distribution allows both peripheral and the central visual field testing with increased spatial resolution within the same examination [16].

In the current study, we investigated whether CVFD in the central 10-degree of the visual field, which is not present at the HFA 24–2 SITA Standard test, can be similarly revealed by the Octopus G1 test (the general 30-degree glaucoma detection program of the Octopus perimeter) and by the HFA 10–2 SITA Standard test, which is designed to separately investigate the central 10° area of the visual field with increased spatial resolution.

## Materials and methods

### Study population

This cross-sectional study was approved by the ethics committees of University of Rome Tor Vergata (Registro Sperimentazioni 43.15) and IRCCS Fondazione G.B.Bietti (Registro Sperimentazioni N.54/17/FB) where the study was conducted in accordance with the Declaration of Helsinki.

Patients were recruited between October 2015 and March 2017 among those referring to the Glaucoma Unit of University of Rome Tor Vergata and to the Glaucoma Unit of IRCCS-Fondazione G.B.Bietti. Patients older than 18 years of age who were able to understand and sign the written informed consent were enrolled. All participants signed the informed consent prior to entering the investigation. Forty-six patients were screened to be included in the study. Five of these patients were excluded because they were not able to perform reliable visual field tests.

The inclusion criteria were the diagnosis of early glaucoma defined with a repeatable HFA 24–2 SITA Standard visual field defect comparable with glaucoma; a Mean Deviation (MD) value ≥ -6 dB; optic nerve head and retinal nerve fiber layer (RNFL) damage which was confirmed with spectral domain optical coherence tomography (Spectralis OCT, Heidelberg Engineering, Heidelberg, Germany). The patients had to have sufficient experience in standard automated perimetry (3 or more reliable tests in the last 2 years). The glaucomatous VF loss had to be confirmed in at least 2 consecutive tests. It was defined with a glaucoma hemifield test result outside the normal limits; a MD and pattern standard deviation with p<0.05 probability of being normal; on the pattern standard deviation plot a cluster of at least 3 contiguous points of p<0.05 which are not contiguous with the blind spot and do not cross the horizontal midline; where 1 of the 3 contiguous points had to have p<0.01 of being normal. The visual fields were classified as reliable if the false positive response rate was <15% and the rate of fixation losses and false-negative responses was <25%.

The exclusion criteria were the presence of a CVFD within the central 10-degree visual field area of the HFA-24-2 test, defined with the presence of a cluster of 3 significantly depressed contiguous points with p<5% in at least 2 test locations, and p<2% in at least 1 test location within the central 12 test point area; history of any other optic neuropathy; any retinal and macular disease; optical media opacities that could have biased the functional and structural test results; spherical refractive error greater than ±6 diopters; and an astigmatism greater than ±3 diopters.

### Clinical examination

In the screening visit all patients underwent a detailed comprehensive ophthalmological examination including the determination of the best corrected visual acuity, slit lamp evaluation, a HFA 24–2 SITA Standard test (only for patients with the last exam more than 3 months prior to the study), an IOP measurement with Goldmann applanation tonometry, and indirect ophthalmoscopy with a 90 diopters lens via dilated pupils. Additionally, all patients had peripapillary RNFL thickness measurements with the Spectralis SD-OCT 3.5 mm circular scan pattern.

A second visit was scheduled within 2 weeks from the screening visit for the eligible patients, in which they underwent a HFA 10–2 SITA Standard test and an Octopus G1 Dynamic program examination on Octopus 900 perimeters using the standard parameters (Goldmann III spot size and 31.5 dB background luminance) in random order. A 15-minute separation was used between the tests. When a visual field result was unreliable according to the criteria listed above, a third visit was scheduled to complete the study. Only eyes with reliable tests were included in the analysis.

### Outcomes

The primary outcome of the study was the comparison of the CVFD detection rates between the HFA 10–2 test and the Octopus G1 program as defined below.

CVFD for both HFA 10–2 and Octopus G1 tests was defined as the presence of a cluster of 3 significantly depressed contiguous points with p<5% on at least 2 test locations and p<2% on at least 1 test location in the whole grid of the pattern deviation plot of the HFA 10–2 test or within the 17 central test point locations of the corrected probability plot of the Octopus G1 program.

The secondary outcome measures comprised the agreement of the test outcomes in detecting the presence of a CVFD; the comparison of test durations; and the comparison of the numbers of depressed test locations outside the central 10-degree visual field area, between the HFA 24–2 test and the Octopus G1 program.

### Statistical analysis

The statistical analysis was performed using JMP Ver. 9.0.1 (SAS Institute Inc.). Normal distribution of the data was investigated with the Shapiro-Wilk test. Since all variables showed a normal distribution, mean ± SD was used for descriptive statistics. Analysis of variance for repeated measures and the paired t-test were used to compare variables. The McNemar test was used to compare the number and percentage of eyes detected with CVFD between the tests and Cohen’s kappa was used to calculate the agreement between test classifications. A p value<0.05 was considered statistically significant.

## Results

Forty-one eyes of 41 patients were included in the study. The demographic data of study population are reported in Table 1.

**Table 1:** Demographic data of study population.

	N = 43 (mean ± SD)
**Age (years)**	65.2±10.1 (range: 38–85)
**Sex (M/F)**	22/19
**BCVA (decimal)**	9.8±0.4
**RNFL Global (μm)**	68.8±11.8
**IOP (mmHg)**	14.7±2.7

SD = standard deviation; M = male; F = female; BCVA = best corrected visual acuity; RNFL = retinal nerve fiber layer measured with optical coherence tomography; IOP = intraocular pressure.

**Fig 1:**
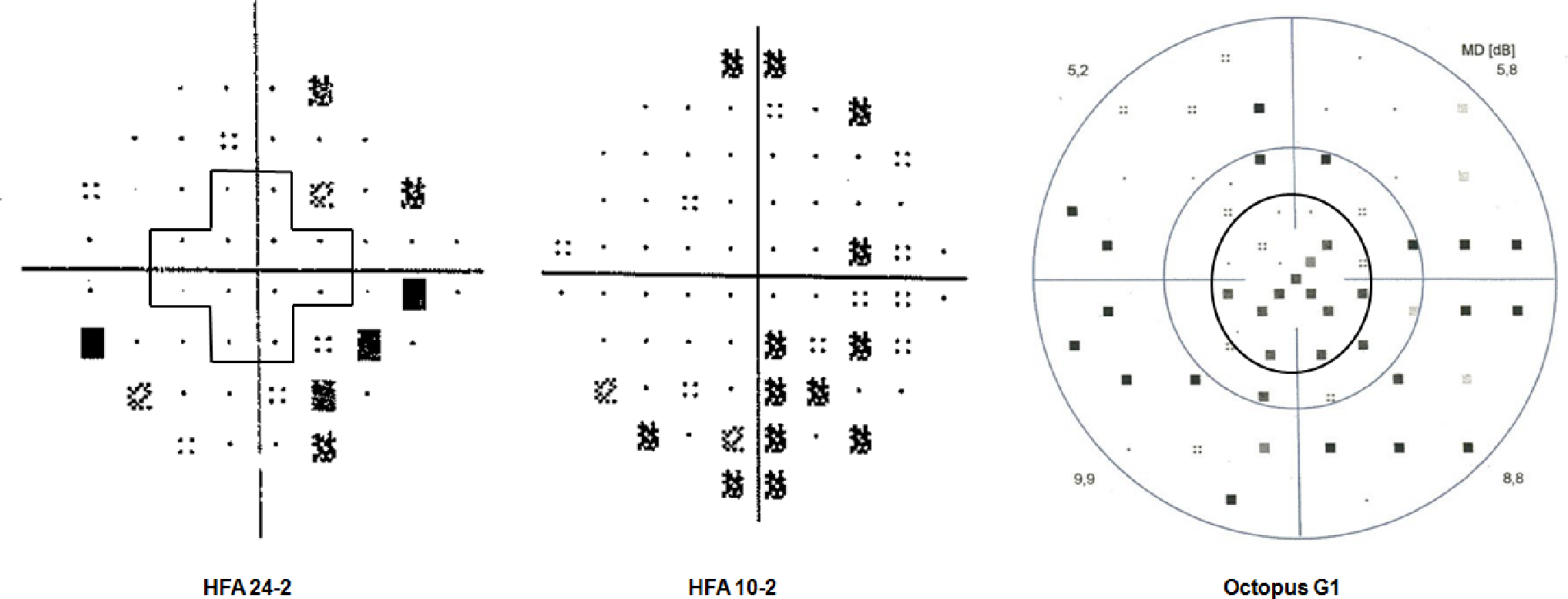
Pattern deviation plots of the Humphrey Field Analyzer (HFA) 24–2 test and the HFA 10–2 test, and the corrected probability plot of the Octopus G1 program of the same early glaucoma eye from the study. The pattern deviation plot of the HFA 24–2 test shows no depressed test point within the central 10° area (the area surrounded by the cross shape line) but the sensitivity is reduced in the more peripheral area of the visual field. The pattern deviation plot of the HFA 10–2 test shows a cluster of depressed test point locations. The peripheral retinal sensitivity is not tested. The corrected probability plot of the Octopus G1 program shows a cluster of reduced sensitivity both in the central 10° area (the region within the inner black circle) and in the periphery.

**Fig 2:**
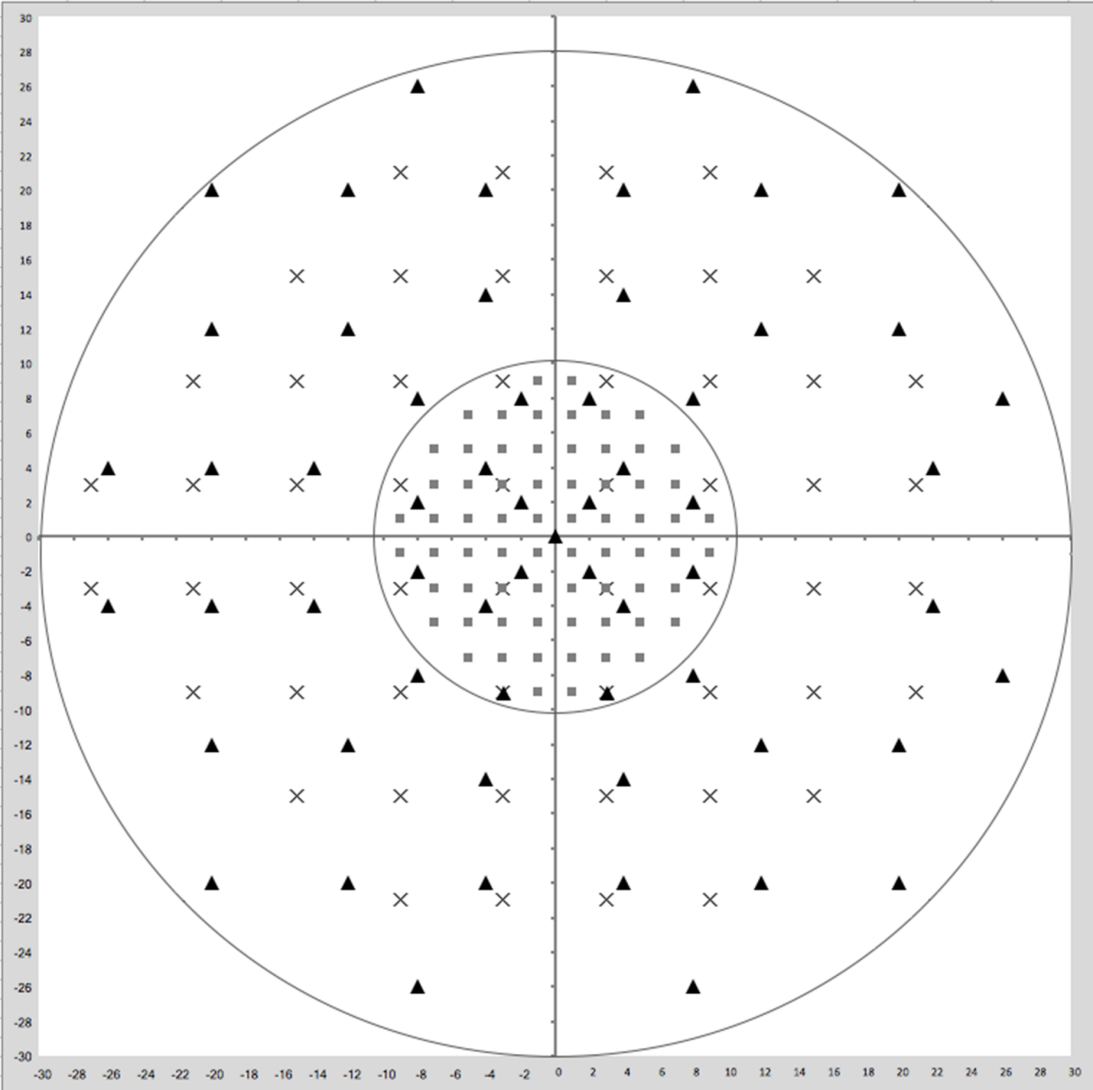
Central 10 degrees of the visual field with the locations of HFA 24–2, HFA 10–2 and Octopus G1 plotted in the same grid. The inner circle delimits the central 10 degrees area with test locations of the three tests (HFA 24–2, HFA 10–2 and Octopus G1) marked with different symbols. Cross = HFA 24–2; square = HFA 10–2; triangle = Octopus G1.

The summary of the three test results is reported in Table 2, and a comparative example of the printouts of the three programs is shown in Fig 1, while Fig 2 shows the central 10 degree of the visual field with the locations of HFA 24–2, HFA 10–2 and Octopus G1 marked with different symbols.

**Table 2:** Visual field results with the HFA 24–2 and HFA 10–2 test and the Octopus G1 program.

	HFA 24–2 (n = 41)	HFA 10–2 (n = 41)	Octopus G1 (n = 41)
**Number of eyes with CVFD**	0	33 (80.4%)	23 (56.0%)
**Test time (min)**	5.5±0.7	5.6±0.8	5.2±1.1
Mean Deviation Humphrey/Mean Defect Octopus (dB)*	-3.26±2.6	-1.67±2.1	4.81±2.8
**PSD/sLV (dB)**	3.84±2.4	2.20±1.9	4.58±1.7
**Number of depressed test locations outside the central 10**^**o**^ **area**	11.95±5.5	N/A	19.68±10.6

PSD = pattern standard deviation; HFA = Humphrey field analyzer; sLV = square loss variance; CVFD = central visual field defect: MS = mean sensitivity; N/A = not applicable.

* In Humphrey visual field analysis, the abnormal mean deviation values are negative numbers; in Octopus perimetry the abnormal mean defect values are positive numbers

CVFD was present in 33 (80.4%) HFA 10–2 test and in 23 (56.0%) Octopus G1 tests (McNemar test, p = 0.002). No eyes without a CVFD on the HFA 10–2 test showed a CVFD on the Octopus G1 program, while the presence of CVFD in 10 HFA 10–2 tests was not matched by the Octopus G1 test results. Thirty-one of 41 eyes were classified as having or not having CVFD, respectively, with both tests (Cohen’s kappa 0.47, Table 3). Considering the HFA 10–2 test as the index test the Octopus G1 program showed a sensitivity of 69.6% (23/33 eyes) and a specificity of 100% (8/8 eyes) to reveal a CVFD that was not present at the HFA 24–2 test.

**Table 3:** Agreement of the classification of the central 10-degree visual field between the HFA 10–2 test and the Octopus G1 test.

		Octopus G1		
		Normal	Abnormal	Total
**HFA 10–2**	**Normal**	**8**	**0**	8
	**Abnormal**	**10**	**23**	33
	**Total**	18	23	41

All eyes (n = 10) with the CVFD detected on the HFA 10–2 but not detected with the Octopus G1 showed visual field defects between 10° and 30° eccentricities.

The number of depressed test point locations (p<5%) outside the central 10°area was significantly higher with the Octopus G1 program (19.68±10.6) than with the HFA 24–2 test (11.95±5.5, p<0.001).

The mean test duration was not significantly different between the tests (5.5±0.7 minutes for the HFA 24–2 test, 5.2±1.1 minutes for the Octopus G1 program, and 5.6±0.8 minutes for the HFA 10.2 test (ANOVA, p = 0.13). The time needed to perform the Octopus G1 program was significantly less (paired t-test, p<0.001) than that needed to perform both HFA tests (11.1±1.3 minutes).

## Discussion

It has been shown that the HFA 24–2 test may underestimate the presence of CVFD due to its low spatial resolution and only 12 test locations in the central 10° of the macular area [8]. Therefore, in the current investigation we compared the ability of the HFA 10–2 SITA Standard test and the Octopus G1 program, Dynamic strategy for the detection of CVFD in early glaucoma cases where the HFA 24–2 SITA Standard test failed to identify CVFD in the central 10-degree area. The background of our investigation was that glaucoma eyes with normal central 10-degree visual field on the HFA 24–2 printout may actually have CVFD [10–15], and this CVFD may be detected by using programs that employ increased spatial resolution in the central macular representation. Such programs are the Octopus G1 program and the HFA 10–2 test. For the Octopus G1 program we used the Dynamic test strategy which is fast and precious in threshold sensitivity determination [16], while for the HFA 10–2 test we used the SITA Standard program which is the most commonly used Humphrey strategy in routine clinical practice [7]. As far as we know this is the first study in which such a comparison was made.

Though CVFD was significantly more frequently found with the HFA 10–2 test than the Octopus G1 test (80.4% vs. 56.0%), the agreement of classification was substantial since 75.6% of the eyes was classified consistently by the two tests. In 10 eyes a CVFD was present at the HFA 10–2 test but not at the Octopus G1 test. Four of these eyes had only one test location with significantly reduced retinal sensitivity at Octopus G1, while six of these eyes had two scattered test locations with significantly reduced retinal sensitivity, failing to meet the cluster criteria applied to define CVFD.

Considering the HFA 10–2 test as the index test the Octopus G1 program had 69.6% sensitivity and 100% specificity for CVFD. The better performance of the Octopus G1 program over the HFA 24–2 test was expected since the former general glaucoma program uses 59 test point locations for the total 30-degree tested area, and of them an increased density grid of 17 test point locations is within the macula representation [16]; in contrast the latter test uses 54 test point locations for a 24-degree visual field area, and only 12 of the 54 test points are located in the central 10° [8]. A higher sensitivity of the HFA 10–2 test over the Octopus G1 program in CVFD detection was also expected since the HFA 10–2 test employs a test-point grid of high spatial resolution due to its 68 test point locations in the central 10-degree visual field [8].

The high specificity of the Octopus G1 program in CVFD detection suggests that the Octopus G1 program probably does not produce many false positive cases in CVFD detection in routine clinical practice.

Our results can be considered as a good trade-off between the time spent with visual field examinations and the efficacy of CVFD detection in early glaucoma. Our results show that in our early glaucoma population, in which the CVFD remained undetected with the HFA 24–2 test which is the typical primary HFA test for glaucoma in routine practice, the Octopus G1 program alone (mean duration 5.6 minutes per eye) produced an approximately 70% sensitivity and 100% specificity in the detection of CVFD compared to the HFA 10–2 test, which was developed for a detailed evaluation of the central 10-degree visual field. Thus, in our early glaucoma population 70% sensitivity and 100% specificity in CVFD detection was achieved in 5.6 minutes with a single Octopus G1 investigation compared to one HFA 24–2 test and one HFA 10–2 test (total mean duration 11.1 minutes). In addition, all eyes with a CVFD detected on the HFA 10–2 printout but not detected with the Octopus G1 program were classified as glaucomatous based on the visual field deterioration between the 10 and 30 degrees eccentricities, and significantly more abnormal test points were detected with the Octopus G1 program in this area. Thus, no early glaucoma case was missed.

The importance of accurate testing the retinal sensitivity in the macular area in all glaucomatous eyes has been highlighted in recent studies exploring the association between quality of life measures, such as NEI VFQ-25 score, and the visual field status as measured with HFA 10–2 and HFA 24–2 tests [17]. The authors reported that the central 10-degree visual field appears to have a stronger association with quality of life than findings on the HFA 24–2 tests, even in the early stages of the disease. Further, a particularly strong association between the HFA 10–2 test results and quality of life was found in those patients that showed low quality of life despite the presence of early HFA 24–2 damage (expressed as mean sensitivity), suggesting that they might have undetected macular damage. The macula comprises approximately 30% of all retinal ganglion cells, and supplies information for 55% to 60% of the primary visual cortex [18]. Thus, it is not unexpected that a damage in the macula can substantially affect the health-related quality of life [17].

Several studies suggest that in order to increase the detection rate of CVFD, an additional test specifically designed to investigate the central macular area should be considered across the whole spectrum of glaucoma severity [10–15]. The HFA 10–2 test comprises 68 locations spaced 2°apart in the central 10°, thus it fulfills the proposed criteria. Hood et al. added two additional points to the HFA 24–2 test pattern to get the same number of points (9 points) in the central 10° of the upper visual field as for the Octopus G program, and compared the ability of the grids (HFA 24–2, Octopus G and HFA 24–2+2 test locations) to detect the average number of abnormal test points with sensitivity ≤ -5dB [19]. They found that the Octopus G program did better than the HFA 24–2 test, but the pattern in which two points were added to the HFA 24–2 test at -1°, 5° and 1°, 5° positions performed even better. The two points were in fact arbitrarily added considering the inferior vulnerable macular region studied in a previous model of glaucomatous damage of the macula [20]. Chen et al collected data on the Medmont M700 perimeter (Medmont International, Nunawanding) and determined which two locations could be added to the central 10° of the 24–2 pattern to improve the detection of macular damage due to glaucoma. They found that pairs of locations in the superior macula region were more often abnormal than pairs in the inferior, thus confirming results of the study by Hood et al [20,21].

Among the limitations of the present study we should highlight that the presence of potential artefacts in terms of false positive and false negative test results cannot be completely ruled out. To try to minimize the presence of false positives and false negatives in our sample population we selected only patients already experienced with visual field testing and to improve the reliability of the detection of CVFD, the cluster criterion for abnormal test locations, rather than less stringent criteria (such as scattered abnormal test locations, or global indexes), was chosen to define CVFDs. So, we might have to speak of "matches/mismatches" rather than defect detections, at least in cases where both protocols had in principle been able to detect a defect. Moreover, differences in the normative databases of the different perimeters under investigation in this work may have contributed to the observed differences in the detection rates of CVFD since the outcome measures chosen to define CVFDs are not absolute measures of retinal sensitivity but probabilities of abnormality estimated against normative databases.

It must be highlighted also that, as reported in Fig 2, while there are 4 overlaying test locations between HFA 24–2 and HFA 10–2, there are no overlaying test locations between Octopus G1 and HFA 10–2. Therefore, it is difficult to determine if the mismatch between HFA 10–2 and Octopus G1 in detecting CVFD is due to the lack of spatial correspondence of test locations of the two grids or is due to the inability of Octopus G1 to detect CVFD detected by HFA 10–2.

Finally, although it was beyond the scope of the present study, the presence of CVFDs was not confirmed in this work by the presence of abnormal retinal biomarkers. Future studies might in fact consider to employ as a confirmation criterion for the presence of CVFD retinal biomarkers such as RNFL defects in the macular vulnerability zone (i.e. narrow infero-temporal region of the circumpapillary retinal nerve fiber layer thickness) or the nasalization of the central retinal vessel trunk location within the optic nerve head [20, 22–24].

The high number of research investigations aiming to increase the ability of the HFA tests to detect visual field deterioration within the central 10-degree area shows the need of better testing the central macular representation. But at the same time in routine clinical practice such efforts are limited by the extra time and cost of routine selective macular testing. Thus, a compromise between sensitivity of CVFD detection and time/cost may also be welcomed in glaucoma clinics. In this respect using one test that evaluates both the central 10-degree area and the total 30-degree visual field for glaucomatous deterioration represents a useful tool. The Octopus G1 program and Dynamic strategy are existing and widely used instruments which together offer a favorable compromise, as shown by the results of the current investigation. We think that our results clarify the ability and benefit of using the Octopus G1 program for the detection of CVFD and peripheral glaucomatous visual field deterioration at the same time. Since this program is available and therefore offers an immediate support to routine glaucoma detection we suggest to consider it for primary visual field investigation in glaucoma detection until more sensitive combined central and peripheral visual field tests become available.
